# Stem cell therapies to modulate harmful immune responses in kidney disease: progress toward clinical validation

**DOI:** 10.1093/stmcls/sxag015

**Published:** 2026-03-28

**Authors:** Qifeng Ou, Fuxuan Li, Shengkun Wang, Ruixue Chen, Cuiqing Ma, Matthew D Griffin

**Affiliations:** Regenerative Medicine Institute (REMEDI) at CÚRAM Research Ireland Centre for Medical Devices, University of Galway, Galway, Ireland; Department of Hand and Microsurgery, Xiangya Hospital, Central South University, Changsha, China; Department of Immunology, Key Laboratory of Immune Mechanism and Intervention on Serious Disease in Hebei Province, Hebei Medical University, Shijiazhuang, China; Joint Stem Cell Research Center, Hebei Medical University-University of Galway, Hebei Medical University, Shijiazhuang, China; Department of Immunology, Key Laboratory of Immune Mechanism and Intervention on Serious Disease in Hebei Province, Hebei Medical University, Shijiazhuang, China; Joint Stem Cell Research Center, Hebei Medical University-University of Galway, Hebei Medical University, Shijiazhuang, China; Department of Immunology, Key Laboratory of Immune Mechanism and Intervention on Serious Disease in Hebei Province, Hebei Medical University, Shijiazhuang, China; Joint Stem Cell Research Center, Hebei Medical University-University of Galway, Hebei Medical University, Shijiazhuang, China; Department of Immunology, Key Laboratory of Immune Mechanism and Intervention on Serious Disease in Hebei Province, Hebei Medical University, Shijiazhuang, China; Joint Stem Cell Research Center, Hebei Medical University-University of Galway, Hebei Medical University, Shijiazhuang, China; Regenerative Medicine Institute (REMEDI) at CÚRAM Research Ireland Centre for Medical Devices, University of Galway, Galway, Ireland; Joint Stem Cell Research Center, Hebei Medical University-University of Galway, Hebei Medical University, Shijiazhuang, China; Discipline of Advanced Therapies, School of Medicine, College of Medicine, Nursing and Health Sciences, University of Galway, Galway, Ireland; Clinical Trials Institute, University of Galway, Galway, Ireland

**Keywords:** kidney disease, kidney transplantation, stem cells, inflammation, immune response

## Abstract

Stem cell therapies hold promise for halting or reversing kidney disease and improving kidney transplant (KTx) outcomes. One route to large-scale clinical application of stem cell therapies for kidney disease is through their capacity to modulate the balance between tissue injury and repair via crosstalk with other cells. Among the key disease-modulating effects of stem cells is their interaction with components of the immune system involved in harmful inflammation during acute kidney injury (AKI), chronic kidney disease (CKD) and complications of KTx. Extensive basic research demonstrates that stem cells employ diverse paracrine mechanisms to re-program immunological activities from pro-inflammatory/pro-fibrotic to anti-inflammatory/pro-repair. The therapeutic benefits of these effects are confirmed in many pre-clinical models of AKI, CKD and KTx for autologous and allogeneic stem cells including hematopoietic stem cells, mesenchymal stem cells, renal progenitor cells, and induced pluripotent stem cells. Nonetheless, translating these findings into therapeutic immunomodulatory cell products that improve the lives of those with kidney disease is highly challenging. The aims of this review are to: (a) Summarize recent insights into the common molecular and cellular mechanisms of immune-mediated tissue injury in kidney disease and KTx along with the types of stem therapies that have been developed to address them. (b) Critically evaluate the extent to which clinical trials of stem cell products have validated such effects in humans with kidney disease and KTx. (c) Identify key bottlenecks to the large-scale application of stem cell therapies to reduce the burden of kidney disease on patients and societies.

## Graphical abstract

**Graphical Abstract:**
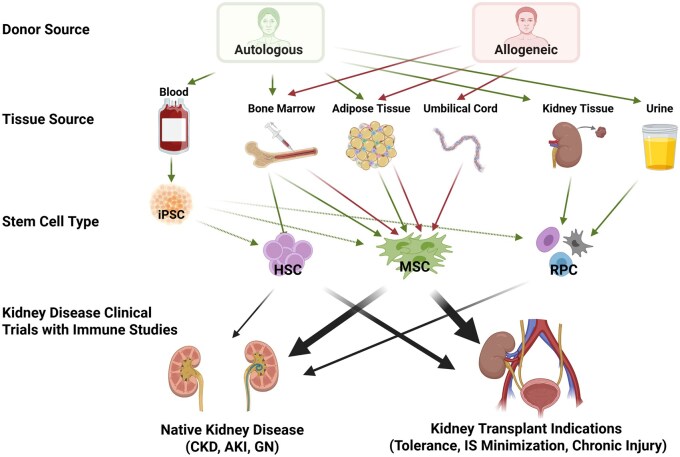

Significance Statement A common feature of many kidney diseases and of kidney transplant complications is the role played by harmful activation of elements of the immune system which drive inflammation, fibrosis and loss of kidney function. Many scientific studies have demonstrated that administration of various types of stem cells results in re-programming of harmful, pro-inflammatory immune responses within the kidney to anti-inflammatory/pro-repair responses which can halt disease progression. This article summarizes and critically reviews the immune modulatory effects of stem cell therapies in the context of kidney disease and the extent to which they have been validated in human clinical trials worldwide.

## Introduction

Kidney disease is a globally significant cause of ill health, premature death and loss of productive life.^1^ The potential to halt or reverse kidney disease through stem cell and regenerative medicine strategies has been recognized for decades. Although stem cell technologies have the potential to directly regenerate functional nephrons or engineer new kidneys by recapitulating renal developmental pathways, such approaches must overcome formidable challenges.^2^ Meanwhile, it has become clear that stem/progenitor cells of diverse types also exert clinically important benefits through complex cross-talk with other cells following introduction into the body.^3^ One form of interaction that has proven to be of high therapeutic relevance to kidney disease and kidney transplantation (KTx) occurs between stem cells and components of the immune system which cause harmful inflammation leading to renal parenchymal fibrosis, atrophy and loss of function. In this review, we: (a) Briefly present current concepts of the roles played by maladaptive immune/inflammatory responses in kidney disease and KTx. (b) Summarize research that has revealed specific types of stem/progenitor cells to exert beneficial modulatory effects on these responses. (c) Summarize and critically evaluate the extent to which the immune modulatory effects of stem cell therapies for kidney disease and KTx have been documented in clinical trials. (d) Reflect on the barriers and bottlenecks that are slowing progress in translating experimental research to large-scale clinical application in this area.

## Dysregulated inflammation and immune responses in kidney disease and transplantation

*Shared and distinct mechanisms of immune-mediated injury in native kidney disease and kidney transplantation:* Kidney disease and complications of KTx involve dysregulated inflammatory and immune responses as central drivers of tissue injury and progressive functional impairment. Activated neutrophils, mononuclear phagocytes (monocytes and macrophages), dendritic cells (DCs), natural killer (NK) cells and other innate lymphocytes, CD8^+^ cytotoxic and CD4^+^ T helper cells, and B cells may infiltrate kidney tissue, promoting acute and chronic inflammation, extracellular matrix deposition, and fibrosis via cell-cell interactions and soluble mediators.^4–6^ In KTx rejection, the combination of innate immune cell activation and donor antigen-specific T cell and B cell responses triggers endothelial injury, complement activation, microvascular inflammation, and, ultimately interstitial fibrosis.^5^^,^^7^ Crucially, regulatory immune cell populations, including regulatory T cells (Tregs), B cells (Bregs), macrophages and DCs play essential roles in limiting immune-mediated damage.^8^^,^^9^ However, these regulatory mechanisms may be impaired or inadequately activated due to immunosuppressive therapies and persistent inflammatory stimuli. Such an imbalance favoring inflammatory and profibrotic mechanisms characterizes both native kidney disease and KTx complications and is a driver of chronic tissue injury and progressive loss of function. Chronic kidney disease (CKD) is increasingly recognized as a state of persistent low-grade inflammation, or “inflammaging”, characterized by elevated systemic levels of pro-inflammatory mediators such as interleukin (IL)-6, tumor necrosis factor (TNF), and C-reactive protein (CRP).^10^^,^^11^ Early in disease progression, monocyte-derived macrophages infiltrate the renal parenchyma and adopt pro-inflammatory phenotypes, releasing reactive oxygen species, nitric oxide, and pro-fibrotic cytokines that drive tubular injury, interstitial inflammation, and extracellular matrix remodeling.^12^ Additionally, NK cells exhibit increased activation and enhanced cytotoxic functions in CKD, contributing to tubular apoptosis and sterile inflammation.^13^ Adaptive immune responses are marked by infiltration of CD8^+^ T cells and CD4^+^ T helper (Th) cells, particularly Th1 and Th17 subsets, secreting interferon-gamma (IFN-γ) and IL-17, respectively, and exacerbating epithelial and endothelial damage.^14^

Kidney transplantation elicits distinct immune responses as the recipient’s immune system responds to donor-derived (allo) antigens, triggering both cellular and antibody-mediated rejection mechanisms.^7^^,^^15^ T cell–mediated rejection (TCMR) involves activation of recipient CD8^+^ cytotoxic T cells and CD4^+^ Th1 cells, which infiltrate the graft and induce endothelial and parenchymal injury through the release of perforin, granzyme, and inflammatory cytokines.^5^^,^^7^ Antibody-mediated rejection (ABMR) is mediated by donor-specific antibodies (DSA) produced by recipient B cells, which bind graft endothelial antigens and activate the classical complement pathway, resulting in microvascular inflammation and characteristic C4d deposition.^7^^,^^16^ Recently, single-cell RNA sequencing has highlighted upregulation of cell-adhesion genes, mechanistically linking interstitial infiltration and tubulitis in TCMR, and peritubular capillaritis and glomerulitis in ABMR.^17^ Additionally, interactions between NK cells (activated via Fc receptor engagement with alloantigen-specific antibodies), CD4^+^ T cells, and graft endothelial cells stimulate the production of pro-inflammatory gene products such as CXCL9, CXCL10, and FCGR3A, further amplifying inflammation.^18^

*Immunological contributions to renal fibrosis and their clinical implications:* The perpetuated inflammation in both CKD and KTx injury commonly culminates in interstitial fibrosis and tubular atrophy (IFTA), a pathological hallmark of irreversible damage. In general, infiltrating leukocytes sustain a pro-fibrotic microenvironment by activating fibroblasts and pericytes via TGF-β, PDGF, and connective tissue growth factor (CTGF) pathways, which induce kidney fibrosis and scarring with concomitantly impaired renal function.^19–21^ Recent advances using single-cell transcriptomics and spatial analyses have further reshaped our understanding of the complex immunological niches within the kidney that drive the progression from injury and acute inflammation to chronic inflammation and fibrosis.^12^^,^^21–24^ For instance, Siglec-F–expressing activated neutrophils enhance renal injury through the production of collagen I and pro-inflammatory cytokines such as TNF and IL-1β.^25^ Monocytes and macrophages recruited by their chemokine receptors such as CCR2, dynamically change phenotypes, initially appearing as pro-inflammatory cells and subsequently transitioning toward anti-inflammatory/pro-repair phenotypes.^12^^,^^20^ CXCL1 secreted by injured tubular epithelial cells can recruit CXCR2-expressing basophils, which also contribute to extracellular matrix (ECM) deposition either directly or indirectly by secreting IL-6, thereby promoting Th17 recruitment and amplifying the tissue remodelling process.^26^ Pericytes further contribute to fibrosis by engaging in aberrant wound-healing responses and producing additional pro-fibrotic factors.^24^^,^^27^ On the other hand, anti-inflammatory immune cells, including Tregs, regulatory macrophages, and tolerogenic (Tol)DCs, have potential to mitigate fibrosis but may fail to do so in a prevailing inflammatory environment.^20^ A common theme to these events is the role played by coordinated immunometabolic reprogramming (shifting from glycolysis during inflammation to fatty-acid oxidation during resolution) as a gatekeeper of regenerative versus fibrotic outcomes in kidney diseases and transplantation.^28^

**Figure 1.**
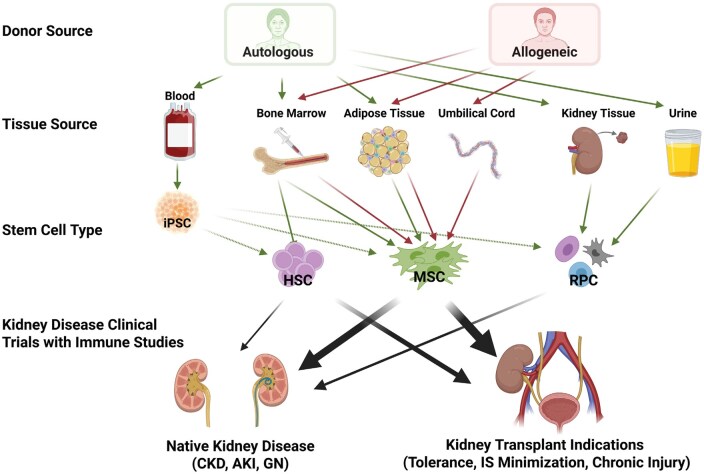
Sources and types of stem cells that have been developed and investigated for native kidney diseases and kidney transplant indications and the extent to which their safety, feasibility, efficacy and immune modulatory functions have been studied through clinical trials in humans to date. Abbreviations: iPSC, induced pluripotent stem cells; HSC, hematopoietic stem cells, MSC, mesenchymal stem cells; RPC, renal progenitor cells; CKD, chronic kidney disease, AKI, acute kidney injury; GN, glomerulonephritis; IS, immunosuppression

**Figure 2.**
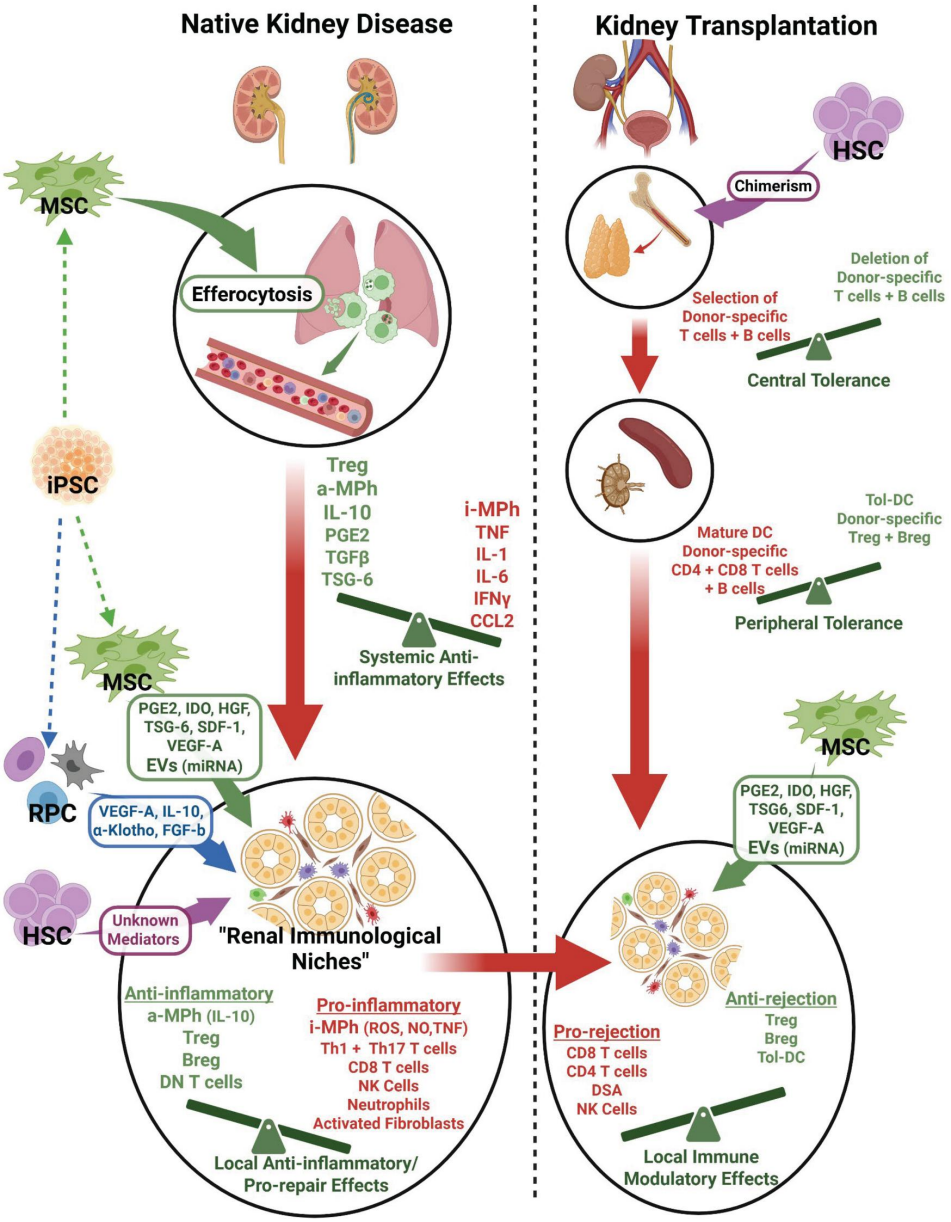
Immunological mediators and niches associated with inflammatory processes in native kidney disease and kidney transplantation and the mechanisms of the immune modulatory/anti-inflammatory effects of stem cell therapies. Abbreviations: MSC, mesenchymal stem cells; iPSC, induced pluripotent stem cells; RPC, renal progenitor cells; HSC, hematopoietic stem cells; Treg, regulatory T cells; aMPh, anti-inflammatory mononuclear phagocytes; iMPh, inflammatory mononuclear phagocytes; IL-10, interleukin 10; PGE2, prostaglandin E2; TGFβ, transforming growth factor beta; TSG-6, tumor necrosis factor-inducible gene 6 protein; TNF, tumor necrosis factor; IL-1, interleukin 1; IL-6, interleukin 6; IFNγ, interferon gamma; CCL2, C-C motif chemokine ligand 2; IDO, indoleamine 2-3 dioxygenase; HGF, hepatocyte growth factor; SDF-1, stem cell derived factor 1; VEGF-A, vascular endothelial growth factor A; EVs, extracellular vesicles; miRNA, microRNA; FGF-b, fibroblast growth factor basic; Breg, regulatory B cells; DN T cells, double negative T cells; ROS, reactive oxygen species; NO, nitric oxide; Th1, T helper type 1; Th17, T helper type 17; NK cells, natural killer cells; DC, dendritic cells; Tol-DC, tolerogenic dendritic cells; DSA, donor-specific antibodies

Given the central involvement of the immune system in both native kidney disease and KTx injury, a range of immune-targeted therapeutic strategies has been developed. These include cytokine blockade (e.g., anti-IL-6 anti-TNF), T cell co-stimulatory inhibitors (e.g., belatacept), B cell–directed agents (e.g. rituximab), and adoptive transfer of regulatory immune cells including Treg, TolDCs, and regulatory macrophages.^29^ While these approaches have shown efficacy in selected contexts, challenges related to tissue specificity, long-term immunosuppression, and therapeutic durability remain. In this therapeutic landscape, stem cell–based therapies have also gained interest for their capacity to modulate immune responses in a more complex manner through paracrine signalling, promotion of regulatory immune phenotypes, and attenuation of inflammatory circuits. Such effects of stem cell-derived therapies hold promise for the preservation and repair of renal epithelial, endothelial, and stromal compartments, collectively reducing fibrotic remodelling and slowing or halting functional decline of both native and transplanted kidneys.^30^ As illustrated in Figure 1, several forms of stem cell therapy have been advanced toward clinical applications in kidney disease on the basis of their efficacy in reducing inflammation, promoting tissue repair, and inducing immune tolerance. The following section of the review describes clinically relevant stem cell therapies for which experimental evidence of beneficial immune modulatory effects have been reported. In each case, known mediators and mechanisms underlying these effects that match our current understanding of the immunopathogenesis of native kidney disease and KTx complications are highlighted. An integrated summary of harmful intra-renal immune responses and their modulation by stem cell therapies is provided in Figure 2.

## Stem cell therapies currently in the clinical pipeline for kidney disease and transplantation

*Stem cell therapies with substantial clinical application (haematopoietic stem cells and mesenchymal stromal cells):* One notable clinical approach has involved the use of hematopoietic stem cell (HSC) transplantation (HSCT) in combination with KTx to achieve mixed lymphohematopoietic chimerism. This approach has shown success in HLA-identical or haplo-identical living donor KTx, with recipients maintaining stable graft function and being able to discontinue immunosuppressive therapy without experiencing rejection or graft-versus-host disease.^31–33^ Mechanistically, this therapeutic approach is primarily ascribed to the development of donor-specific central immune tolerance through seeding of the recipient thymus with donor HSC-derived antigen presenting cells resulting in clonal deletion of donor-responsive T cells–although an element of peripheral tolerance through Treg induction may subsequently occur (Figure 2).^33^ Autologous (auto) HSCs (CD34^+^ cells), delivered via intra-renal arterial injection, has also shown preliminary evidence of improving renal function in CKD.^34^ Pre-clinical studies suggested that CD34^+^ HSCs may ameliorate CKD through anti-inflammatory and vasculogenic effects, although specific mediators were not identified.^35^

Mesenchymal stromal cells (MSCs) of autologous (auto) or allogeneic (allo) origin derived from various tissue sources [e.g. bone marrow (BM)-, adipose (AD)-, umbilical cord (UC)] have been extensively investigated for their ability to modulate the immune response and reduce fibrosis in CKD, acute kidney injury (AKI) and KTx. Pre-clinical studies have consistently demonstrated that MSCs attenuate inflammation, promote angiogenesis, reduce fibrosis, and enhance renal repair mechanisms, although their long-term efficacy remains under investigation.^36^ It has come to be broadly accepted that the primary therapeutic mechanisms of action of MSCs in both CKD and AKI rely on paracrine mechanisms such as inducible release of soluble anti-inflammatory/pro-repair mediators and extracellular vesicles (EV), as well as the re-programming of mononuclear phagocytes to anti-inflammatory phenotypes following engulfment of apoptotic MSCs (efferocytosis).^30^ From the many pre-clinical studies that have contributed to the evidence of a paracrine model of MSC therapeutic effects in kidney disease, a number of specific molecular mediators have been repeatedly identified (Figure 2). Those directly secreted by MSCs under inflammatory conditions include prostaglandin E2 (PGE2), hepatocyte growth factor (HGF), kynurenine produced by the action of indoleamine 2,3 dioxygenase (IDO) on tryptophan, tumor necrosis factor-inducible gene 6 protein (TSG-6), stromal cell-derived factor 1 (SDF-1/CXCL12) and vascular endothelial growth factor A (VEGF-A).^30^^,^^37^ Other therapeutically beneficial biomolecules may be transferred to target cells within the kidneys via MSC-released EVs. In this case, specific anti-fibrotic effects have been described for micro (mi)RNAs such as miR-let6c.^38^^,^^39^ Furthermore, the potent anti-inflammatory/pro-repair effects of MSC efferocytosis may be mediated by release of soluble mediators including interleukin (IL)-10, PGE2 and transforming growth factor β (TGF-β) by monocytes and macrophages.^40^ Promotion of Treg through both contact-dependent pathways and soluble mediators (e.g. IL-10) has also been repeatedly linked with the disease-modulating effects of MSCs.^41^ Finally, single cell and spatial transcriptomic analyses of kidney disease models are now providing a more holistic understanding of the pro-inflammatory and pro-fibrotic niches that drive progressive renal injury and of the mechanisms by which MSCs modulate cross-talk among immune cells, tubular epithelial cells and fibroblasts within these niches.^12^^,^^21^^,^^24^^,^^42^ Despite the wide range of potential mediators and pathways, it is implicit in this “paracrine model” that administered MSCs are short-lived (i.e. days to weeks) within the recipient and rely on a legacy of re-programming effects on other cells types for a durable therapeutic benefit.^43^ Nonetheless, the extent to which locally delivered MSCs may engraft for longer periods within the kidneys under some circumstances remains a topic of interest.^44^ It is also worth noting that MSCs also have the capacity to initially trigger innate immune activity through their expression of tissue factor and pattern recognition receptors.^45^ Thus, strategies to manipulate MSCs or MSC-derived EVs to improve their targeting efficiency, tissue persistence or therapeutic potency are of significance to their future clinical applications in kidney disease.^46^^,^^47^

*Stem cell therapies with limited or no clinical application (renal progenitor cells and pluripotent stem cells)*: There has been substantial research interest in adult kidney-derived stem/progenitor cells in the past two decades as well as some controversy as to their identity, significance and therapeutic potential for repair and regeneration of injured/diseased kidneys.^48^^,^^49^ Such cells have been identified from multiple renal microanatomic niches on the basis of cell cycling properties as well as expression of surface proteins, in particular CD24, CD133 and CD106 (VCAM-1).^48^^,^^50^ Isolation and culture expansion of human renal progenitor cells (RPCs) has proven possible from kidney tissue samples (biopsies or nephrectomy specimens) as well as from urine, raising the possibility of clinical applications for kidney regeneration and repair.^48^^,^^51^ The primary therapeutic concept for RPCs arose from their potential to engraft and differentiate into renal parenchymal cells (e.g. tubular cells, podocytes, interstitial fibroblasts) following direct implantation into or migration to diseased kidneys.^52–54^ Nonetheless, in vitro studies and administration of human RPCs to animal models of AKI have also provided evidence for paracrine immune modulatory/anti-inflammatory effects (Figure 2).^52^^,^^53^^,^^55^^,^^56^ Some of the paracrine mediators and modulatory effects of the RPCs overlap with those reported for MSCs–examples being secretion of IL-10, VEGF-A^53^ and modulation of Treg.^55^ Others, however, may be more distinct to RPCs and include secretion of pro-repair factors such as fibroblast growth factor basic (FGF-b), platelet-derived growth factor (PDGF),^53^ and α-Klotho^57^ as well as cytokine cocktails that promote immune regulatory double negative T cells.^55^

Similarly, the application of pluripotent stem cells, in particular induced pluripotent stem cells (iPSCs), to kidney disease and kidney transplantation has captured the interest of translational renal scientists, clinicians and innovators. To date, research in this area has focused heavily on the use of iPSCs to develop kidney organoids for “disease in a dish” modelling or with a view to regeneration of functional kidney tissues for implantation.^58^ Nonetheless, there is also now a body of experimental evidence that human iPSCs or iPSC-derived RPCs and MSCs can modulate acute and chronic kidney disease progression through anti-inflammatory/pro-repair mechanisms.^59^^,^^60^ In a recent example, Araoka et al. demonstrated that localized delivery of iPSC-derived nephron progenitor cells to the kidney ameliorated the severity of CKD and AKI in mouse models through paracrine effects mediated by VEGF-A.^61^ Overall, however, while the potential for RPC- and iPSC-based therapies to be manufactured at scale and to promote kidney repair through modulation of harmful immune responses has been established, the extent to which they have been clinically translated to date is limited.^48^ One exception has been the progenitor-like “selected renal cell” population which was initially demonstrated to mediate regenerative effects following intra-renal implantation in animal models of CKD through paracrine mechanisms^62^ and, as discussed in more detail below, has subsequently shown promising results in early phase clinical trials as a personalised, kidney biopsy-derived cell therapy for diabetic kidney disease (DKD).^63^^,^^64^

## Clinical trial results and evidence of immune modulation

In this section of the review, the extent to which stem cell therapies for kidney diseases have progressed to clinical trials is summarized, highlighting evidence garnered from these trials that such therapies modulate harmful immune responses in human subjects. Table 1 provides summaries from clinical trials which have interrogated the immunological effects of stem cell therapies in native kidney diseases and KTx respectively along with brief comments on the strength of evidence.

**Table 1.** Summary of clinical trial evidence of modulation of immune responses in clinical trials of stem cells therapies for native kidney diseases and kidney transplant indications (excluding hematopoietic stem cells).

Therapeutic Target	Stem cell type (Product name)/Route (dose no.)	No. treated	Control group (No.)	Key efficacy outcomes for stem cell therapy	Immunological studies performed	Key immunological results (compared to controls)	Comments on strength of evidence for immune modulation ^‡^
*Native Kidney Diseases*							
*Diabetic Kidney Disease^66^*	Allo-MPC^†^ (rexlemestrocel-L)/IV (1)	20	Placebo (10)	Trend of reduced rate of GFR decline	Serum inflammatory markers, anti-HLA antibodies	Trend of lower serum IL-6 Transient anti-HLA in 1/20	Two time-points Mechanistically relevant *Single analyte significant at higher dose only*
*Diabetic Kidney Disease^67^*	Allo-BM-MSC (ORBCEL-M)/IV (1)	16	Placebo (4)	Reduced rate of estimated GFR decline	PBMC flow cytometry, serum inflammatory markers, anti-HLA antibodies	Preserved total + memory Treg, reduced NKT cells, and stabilized intermediate monocytes No differences in inflammatory markers Low-level anti-HLA in 1/16	Multi-timepoint monitoring Mechanistically relevant *Significant changes occur at varying time-points* *Limited number in placebo group*
*Renovascular Disease^69^*	Auto-AD-MSC/IA (1)	14	Standard therapy (14)	Increased cortical perfusion, reduced renal hypoxia, stable single-kidney GFR	Renal vein blood angiogenic and inflammatory cytokines	Reduced renal vein VEGF-C after MSC therapy Trend of reduced inflammatory cytokines	Localised sampling Two time-points Mechanistically relevant *Single analyte significant*
*Renovascular Disease^68^*	Auto-AD-MSC/IA (1)	21	Standard therapy (18)	Increased renal blood flow, reduced renal hypoxia, increased GFR, reduced BP	Renal vein blood angiogenic and inflammatory cytokines	Reduced renal vein angiogenic cytokines, NGAL, and IFNγ after MSC therapy	Localised sampling Two time-points Multiple significant results Mechanistically relevant
*Steroid-dependent nephrotic syndrome^79^*	Auto-BM-MSC/IV (2)	18	None (compared to year prior to stem cell therapy)	Longer time to relapse and number of relapses (children only)	PBMC flow cytometry	Increase in Treg, increased transitional B cells, reduced mature + memory B cells	Multi-timepoint monitoring Mechanistically relevant *Significant changes occur at varying time-points*
*Kidney Transplantation*							
*Immunuosuppression minimization or withdrawal^82–85^*	Auto-BM-MSC^†^/IV (1)	4	Historical control KTx recipients	Successful reduction of withdrawal of immunosuppression, stable long-term function	PBMC flow cytometry, anti-HLA antibodies, donor-specific T cell responsiveness	Prolonged high Treg/CD8 T cell ratio, increased transitional B cells, prolonged donor-specific T cell hypo-responsiveness	Multi-timepoint monitoring Extensive profiling and functional assays Mechanistically relevant *Limited subject numbers*
*Immunosuppression reduction^86^^,^ ^88^^,^ ^89^*	Third-party Allo-BM-MSC/IV (2)	10	None	Successful reduction of immunosuppression, stable transplant function	PBMC flow cytometry, anti-HLA antibodies, blood pro- and anti-inflammatory cytokines	No notable changes to major immune cell subtypes Increase in T and B cell activation profile and MSC donor-derived cfDNA and reductions in blood cytokine levels 4 hours after MSC No de novo anti-HLA against MSC or KTx donors	Multi-timepoint monitoring Extensive profiling and functional assays Multiple significant results Mechanistically and temporally relevant *Limited subject numbers* *No control group*
*Immunosuppression reduction^87^*	Auto-BM-MSC/IV (2)	29	28	Successful reduction of immunosuppression, similar transplant function and complication rate	PBMC flow cytometry, anti-HLA antibodies	Increased memory Treg number Higher rate of development of donor-specific anti-HLA	Multi-timepoint monitoring Control group comparison at all timepoints Mechanistically relevant *Significant differences occur at varying time-points*
*Immunosuppression reduction^91^*	Third-party Allo-BM-MSC/IV (1)	10	10	Partial success in immunosuppression reduction, similar transplant function and complication rate	PBMC flow cytometry, anti-HLA antibodies, kidney biopsy immune-staining for T cells	Increased total and activated Treg proportions in blood with no difference in kidney biopsy Treg 40% de novo anti-HLA against MSC or KTx donors	Multi-timepoint monitoring Control group comparison at all timepoints Mechanistically relevant *Significant differences occur at varying time-points*
*Immunosuppression reduction (pilot and follow-up trials) ^92^^,^ ^93^*	KTx donor-derived Allo-BM-MSC/IA + IV (2)	6/16	6/16	Successful reduction in immunosuppression, similar transplant function and complication rate	PBMC flow cytometry, intracellular cytokines, donor-specific T cell responsiveness, chimerism detection	No clear evidence of immune cell modulation, donor-specific hypo-responsiveness or donor chimerism	*No significant evidence of immune modulation despite extensive profiling and functional assays*
*Subclinical acute rejection treatment^97^*	Auto-BM-MSC/IV (2)	6	None	Resolution of acute rejection on repeat biopsy in 2/6 Opportunistic infections in 3/6	PBMC flow cytometry, donor-specific T cell responsiveness	Reduced donor-specific T cell responsiveness following MSC infusions in 5/6 No changes in major immune cells subtypes	Significant results for mechanistically relevant functional assays compared before and after transplant *Limited subject number* *No control group*
*Chronic active ABMR* ^98^	Auto-BM-MSC/IV (3)	3	None	No clinical or histological improvement Severe systemic illness and allograft loss in 1/3	PBMC flow cytometry, blood pro- and anti-inflammatory cytokines	No clear evidence of immune cell or cytokine modulation	*No significant evidence of immune modulation*
*Chronic active ABMR* ^99^	Third-party Allo-BM-MSC/IV (4)	7	Historical controls	Stabilization of eGFR decline, reduced proteinuria	PBMC flow cytometry, anti-HLA antibodies, T cell responses to third party antigens	Trend of increased Treg after MSC infusions Reduced titers of anti-donor HLA antibodies	Multi-timepoint monitoring *Non-significant results without control group comparison* *Limited subject numbers*
*Chronic ABMR* ^100^	Third-party Allo-BM-MSC/IV (1 or 4)	23	10	Slower rate of eGFR decline, superior transplant survival	PBMC flow cytometry, anti-HLA antibodies, blood pro- and anti-inflammatory cytokines	Trend of increased PD1+ CD4 T cells and reduced memory-like B cells Reduced titers of anti-donor HLA antibodies Reduced pro-inflammatory cytokines/chemokines	Multi-timepoint monitoring Extensive profiling assays Changes in multiple analytes *Not all results mechanistically relevant* *Immune assays not compared with control group*

† Abbreviations: Allo-MPC, allogeneic mesenchymal progenitor cell; IV, intravenous; GFR, glomerular filtration rate; HLA, human leukocyte antigen; IL-6 = interleukin 6; IA, intra-arterial; PBMC, peripheral blood mononuclear cell; Treg, regulatory T cells; NKT cells, natural killer T cells; Auto-AD-MSC, autologous adipose-derived mesenchymal stem cell; NGAL, neutrophil gelatinase-associated lipocalin; IFNγ, interferon gamma; Auto-BM-MSC, autologous bone marrow-derived mesenchymal stem cell; KTx, kidney transplantation; PBMC, peripheral blood mononuclear cells; cfDNA, cell free DNA; ABMR, antibody-mediated rejection; eGFR, estimated glomerular filtration rate.

‡ Positive aspects of the evidence provided by each study or studies are in standard text, weaker aspects are in italics.

*Stem cell therapy trials in chronic kidney disease:* Diabetic kidney disease is associated with chronic systemic and intra-renal inflammation and has been the target for a large body of pre-clinical investigation into the potential benefits of immune modulatory stem cell therapies.^30^^,^^65^ Two early phase, placebo-controlled clinical trials have provided promising evidence that singe IV infusions of specific, BM-derived allo-MSC-like products (Rexlemestrocel-L and ORBCEL-M) are safe and may slow the decline of estimated glomerular filtration rate (eGFR) in progressive DKD.^66^^,^^67^ Importantly, both trials also provided correlative immunological data suggesting reduction of pro-inflammatory cytokines (IL-6)^66^ or modulation of the balance between pro- and anti-inflammatory immune cell types (CD16^+^ monocytes and Tregs)^67^ in subjects treated with cells compared to placebo.

Other forms of CKD have also been the subject of stem cell therapy trials. Notably, renal arterial infusion of auto-AD-MSCs in subjects with renovascular disease (RVD) at the Mayo Clinic, USA was shown in two early phase trial cohorts to be associated with increased renal cortical perfusion, decreased renal hypoxia, stabilization of single-kidney eGFR and reduced blood pressure compared to medically treated control subjects.^68^^,^^69^ Associated with these favorable trends were reductions in renal vein levels of inflammatory and angiogenic cytokines^68^—consistent with pre-clinical evidence of anti-inflammatory/pro-repair mechanisms of action reported by the same investigators in a large animal model of RVD.^70^ Of interest, the same group recently reported stabilization of rate of eGFR decline in a patient with progressive DKD following the same protocol of renal arterial infusion of auto-SD-MSCs.^71^

An allo-AD-MSC product (ELIXCYTE) was also recently reported to be associated with stabilization or increase in eGFR in 12 subjects with CKD of varying etiologies following single IV infusions. In this trial, however, no control arm or mechanistic investigations were included.^72^ Ohtake et al. provided preliminary evidence that two autologous CD34^+^ cell infusions into the renal arteries stabilized kidney function in four patients with progressive CKD, likely through immunomodulation and tissue repair, although no mechanistic studies were performed.^34^ Similarly, Carstens et al. reported the outcomes of 18 patients with Mesoamerican nephropathy who received single renal arterial infusions of MSC-rich adipose stromovascular fractions (SVF) cells.^73^ Subjects with an initial eGFR >30 ml/min exhibited stabilization of function over a subsequent 36-month follow-up while those with initial eGFR <30 ml/min did not. A paracrine, anti-inflammatory mechanism of action of SVF cells was proposed but markers of inflammation were not evaluated.^73^ In autosomal dominant polycystic kidney disease, Makhlough et al. observed a reduction in the rate of decline of eGFR in six subjects following IV administration of auto-BM-MSCs.^74^

A novel cell therapy approach for CKD, involving direct implantation of the previously mentioned autologous, kidney biopsy-derived “selected renal cell” product (now referred to as rilparencel), has been investigated in early phase clinical trials.^63^^,^^64^ Mechanistically, this ex vivo cultured cell product is reported to function through pro-regenerative/anti-inflammatory paracrine mediators released or induced by epithelial cells which have reverted to a stem/progenitor-like phenotype.^63^^,^^64^^,^^75^ Results from early phase trials of multiple injections of this therapy have been published for five adults with CKD due to congenital abnormalities of the kidney and urinary tract^76^ and for 53 adults with DKD due to type 2 diabetes.^63^^,^^64^ In these reports, feasibility and safety were demonstrated and, in the case of DKD recipients, post-therapy rate of decline of eGFR was reduced compared to the rate prior to therapy.^63^^,^^64^^,^^76^ A larger, uncontrolled Phase 3 trial in adults with DKD is also underway.^64^ Although extensive animal model experiments and cellular and molecular characterization of the cell product have provided a strong evidence for the proposed pro-regenerative mechanisms of action,^62^ mechanistic studies in clinical trial participants have not been reported to date.

*Stem cell therapy trials in acute kidney injury and glomerulonephritis:* Two other areas of native kidney disease for which clinical trials of stem cells with proposed anti-inflammatory activity have been conducted are AKI and glomerulonephritis (GN). In the case of AKI, Swaminathan et al. reported a negative outcome for the ACT-AKI randomized, double-blind, placebo-controlled clinical trial of intra-aortic infusion of the allo-BM-MSC product AC607 involving 156 adults with early AKI following cardiac surgery.^77^ Although the role of inflammation in post-cardiac surgery AKI is discussed by the authors, the trial did not include analysis of inflammatory biomarkers or other mechanistic pathways. A single case report from a clinical trial of renal arterial infusion of auto-CD34^+^ cells in severe AKI has described improved kidney function in association with a transient increase in inflammatory markers (IL-6, IL-8, CRP) 5 days after cell therapy.^78^ However, no further report from this trial has emerged.

Few trials of stem cell therapies in GN have been completed. Vivarelli et al. have reported detailed results from an open-label clinical trial of two IV infusions of auto-BM-MSCs in 10 children and six adults with corticosteroid-dependent nephrotic syndrome. Although all subjects experienced relapse of nephrotic syndrome following corticosteroid withdrawal, MSC infusion was associated with delayed time to relapse of nephrotic syndrome and reduced number of relapses compared to the prior year in children.^79^ Immune profiling studies revealed transient increases in circulating transitional (potentially regulatory) B cells and Treg in children at 3 to 6 months after MSC infusion without other major effects on immune cell and inflammatory parameters.^79^ Finally, the recently published results of an uncontrolled Phase 1 trial of one or two IV infusions of an allo-AD-MSC product (ADR-001) in 9 adults with IgA nephropathy documented reduced microhematuria and transient reduction in urine protein and urine L-fatty acid binding protein shortly after cell therapy without evidence of lasting clinical remission.^80^

*Stem cell therapy trials of immune tolerance or immunosuppression minimization in kidney transplantation:* A limited number of stem cell therapy trials in KTx have pursued the goal of achieving complete donor-specific immune tolerance. In one of the most important achievements of stem cell therapies, two groups from the US demonstrated, in 2008, that a combination of KTx and HSCT from the same donor can induce central and peripheral immune tolerance through mixed chimerism and can achieve drug-free transplant survival with low rates of graft versus host disease and rejection.^32^^,^^33^ This approach has since been reproduced in other centres.^31^^,^^81^ For example, Wang et al. recently reported favorable long-term outcomes for 10 of 11 living donor KTx recipients who underwent a protocol combining donor HSCT with total lymphoid irradiation and anti-thymocyte globulin to induce immune tolerance.^81^ While such protocols have been demonstrably successful, they remain limited by the extent of the conditioning regimens required to achieve chimerism and by restriction to low immunological risk, living donor transplants. For this reason, the majority of clinical trials of non-HSCT stem cell therapies in KTx recipients have sought to modulate immune responses through other mechanisms with the aim of achieving reduction, minimization or withdrawal of immunosuppressive drugs.

Auto- and allo-MSC therapies have been quite extensively investigated for this purpose and, importantly, clinical trials of MSCs in KTx have frequently included investigation of immunological and inflammatory mechanisms (Table 1). For example, a group from the Mario Negri Institute, Italy have described early and long-term outcomes coupled with detailed longitudinal immunological profiling for a small series of living donor KTx recipients treated with IV infusions of auto-BM-MSCs shortly before or after transplantation for the purpose of facilitating reductions or complete withdrawal of immunosuppression.^82–85^ Key clinical observations to date have included very stable long-term KTx function and achievement of varying degrees of immunosuppressive drug withdrawal–in some cases associated with reversible acute rejection or development of DSA.^82^^,^^83^^,^^85^ Immunologically, some MSC-treated KTx recipients exhibited prolonged high Treg/CD8^+^ T cell ratio, increased transitional B cell number, and reduced donor-specific cellular cytoxicity.^82^ Complete withdrawal of immunosuppression with a continued pro-tolerogenic immunological profile was achieved in one patient.^84^

The group from Leiden University Medical Center, the Netherlands, have pursued similar goals for KTx recipients receiving post-transplant IV infusions of either third-party allo- or auto-BM-MSCs.^86–89^ In the Neptune trial, 10 living donor KTx recipients received two IV infusions of HLA-selected allo-BM-MSCs at 24 and 26 weeks post-transplant followed by minimization of tacrolimus levels in an uncontrolled trial design.^86^ Clinical outcomes at 1 year post-transplant were excellent with no rejection episodes. Longitudinal immunological profiling during the first post-transplant year did not reveal any prolonged modulations of regulatory or other immune cell populations. However, increases in a cluster of activation markers (CD11b, CD11c, CD38, CD39 and Ki67) on circulating T cell and B cell populations^89^ coupled with an observed peak in MSC donor-derived circulating cell free DNA 4 hours after MSC infusion^88^ suggested an early burst of immunological activity associated with MSC programmed cell death and efferocytosis in the lungs.^90^ In the TRITON trial, an open-label randomized controlled design compared 29 living donor KTx recipients that received IV infusions of auto-BM-MSCs at 6 and 7 weeks post-transplantation followed by tacrolimus withdrawal with 28 control recipients maintained on tacrolimus therapy.^87^ Tacrolimus withdrawal was achieved in the majority of MSC recipients and six-month outcomes were comparable between the two groups. Although longitudinal immunological profiling indicated higher numbers of circulating memory Treg in MSC recipients at 6 and 12 months post-transplant, the frequency of DSA development was higher in MSC recipients (24% vs 7%). Nonetheless, an ad hoc follow-up analysis to 5 years showed them to have numerically higher graft survival and eGFR and lower number of biopsy-proven immune-inflammatory complications.^87^

Erpicum et al. reported the results of an open label Phase 1/2 clinical trial of a single IV infusion of third-party allo-BM-MSCs administered 2–5 days after deceased donor KTx.^91^ The 10 MSC-treated recipients received a standard immunosuppressive drug regimen with attempted corticosteroid withdrawal and were compared to 10 control KTx recipients receiving the same immunosuppressive regimen. Transplant survival, eGFR and frequencies of opportunistic infections and rejection episodes at 1 year were similar between the groups. Longitudinal immunological profiling revealed increases in Treg at days 30 and 180 after KTx in MSC-treated recipients. However, corticosteroid withdrawal was only achieved in 30% of MSC recipients and low-level DSAs against MSC donor and/or KTx donor were detected in 40%.^91^ Three centers in China have also described results of early phase controlled clinical trials of MSCs administered around the time of KTx with the aim of reducing immunological complications or facilitating lower immunosuppressive therapy.^92–95^ In the largest of these, 106 living donor KTx recipients that received two sequential IV infusions of auto-BM-MSCs experienced lower rates of both opportunistic infections and acute rejection than a control group induced with anti-IL2R antibody.^95^ This trial did not, however, incorporate immunological monitoring. The two other trials involved administration of allo-MSCs derived from the KTx donor^92^^,^^93^ or from third party donors^94^ delivered as combined intra-arterial infusion during surgery and IV infusions before or after surgery with^92^^,^^93^ or without^94^ subsequent reduction in immunosuppressive drug levels. Outcomes for MSC-induced recipients were generally similar to those of control KTx recipients. The group from Guangzhou performed longitudinal immunological monitoring, mixed lymphocyte reactions and chimerism assays with no evidence of immune modulation or donor-specific tolerance in MSC-induced KTx recipients.^92^^,^^93^ Finally, Vanikar et al. reported results of a three-arm, tolerance induction trial (*n* = 95 per arm) in which donor-derived allo-AD-MSCs and HSCs, HSCs alone or no stem cells were infused into the portal vein at the time of living donor KTx with the aim facilitating immunosuppression withdrawal. Compared with the non-stem cell induced control group, the MSC/HSC- and HSC-induced control groups achieved favorable clinical outcomes for up to 7 years with reduced immunosuppression achieved in >60%. However, frequency of DSA development was high in the stem cell treated groups.^96^

*Stem cell therapy trials of treatment of immunological complications of kidney transplantation:* A limited number of early phase trials have been reported for the application of stem cell therapies to treat immune/inflammatory complications of KTx. In 2013, the Leiden group performed a pilot clinical study of auto-BM-MSCs given as two IV infusions to six KTx recipients with subclinical acute rejection and/or IFTA diagnosed on protocol biopsies taken four weeks and six months post-transplant. Resolution of acute rejection and ITFA was demonstrated in follow-up biopsies and reductions in donor antigen-specific T cell proliferation in immunological assays were observed in some MSC recipients. However, 3/6 also experienced an opportunistic infection, raising the concern of a non-specific immunosuppressive effect of MSC infusions.^97^

Večerić-Haler et al. reported results of a Phase 1/2 clinical trial to evaluate the effects of three sequential IV infusions of auto-BM-MSCs in KTx recipients with chronic active ABMR. Notably, the trial was discontinued following treatment of three patients without evidence of clinical or histological improvement of ABMR and with a severe systemic illness and acute loss of KTx function following the third MSC infusion in one case.^98^ Furthermore, immunological monitoring suggested increases in circulating activated T cells and reduced Treg following MSC infusions. In contrast, Park et al. reported stabilization of eGFR decline, reduced proteinuria and a trend of increasing circulating Treg in the six months after four sequential IV infusions of third-party allo-BM-MSCs in seven KTx recipients with chronic active ABMR.^99^ In the setting of chronic (non-active) ABMR, Wei at al. described favorable results of a clinical trial of one or four consecutive IV infusions of third-party allo-BM-MSCs in 23 KTx recipients. Compared to 10 control recipients with chronic ABMR, MSC-treated recipients exhibited better two year transplant survival, slower rate of eGFR decline and reductions in serum DSA titres and pro-inflammatory cytokine/chemokine.^100^ Furthermore, peripheral blood immune cell profiling documented increases in CD4^+^/PD1^+^ T cells and decreases in IgD^−^/IgM^−^ memory-like B cells during the four weeks after MSC administration.^100^ Finally, in biopsy-proven IFTA not associated with DSA, Zhang et al. treated 11 KTx recipients with one intra-arterial infusion followed by two IV infusions of auto-BM-MSCs.^101^ At 12 month follow-up, transplant function and IFTA score generally remained stable or improved in treated patients. However, no control group or immunological studies were included.^101^

## Bottlenecks to the large-scale translation of stem cell therapies for inflammatory kidney diseases and kidney transplantation

As is clear from the preceding section, there has been a substantial number of early phase clinical trials of stem cell therapies with proposed immune modulatory and anti-inflammatory mechanisms of action in native kidney disease and KTx. These have encompassed varying subject numbers (<10 to >100) and rigor of design (randomized, double blind, placebo controlled to open label, uncontrolled). Furthermore, considering the range of clinical targets for which trials of stem cell therapies were conducted, there have been some contrasting or conflicting results to date. For example, positive signals of clinical benefit have been reported from multiple trials in DKD,^64^^,^^66^^,^^67^^,^^71^ while promising pre-clinical results for AKI have, thus far, not been replicated in clinical trials.^77^ In the area of KTx, there has been relatively consistent success reported from trials of MSC products for the outcome of reduced immunosuppression,^82^^,^^86^^,^^87^^,^^91^^,^^92^^,^^95^ while trials of MSCs to treat acute and chronic immunological complications of KTx have yielded mixed signals.^97^^,^^98^^,^^100^ Among the trials that specifically included analyses of immunological and inflammatory processes, some but not all have provided encouraging signals of distinct modulations of immune responses. The most frequent observations reported have been increased circulating Treg, modulation of the B cell repertoire, and reduction of pro-inflammatory mediators that likely reflected stem cell-induced anti-inflammatory immune responses. Notably, however, the strength of evidence from individual trials for mechanistically relevant immune modulatory effects of stem therapies for both native kidney disease and KTx indications, remains relatively modest (Table 1).

Complicating the interpretation of clinical trial results further is the variable use of autologous and allogeneic cell therapies. In the case of KTx indications, an additional layer of complexity is introduced by the potential for allogeneic stem cells to be derived from either the kidney donor (“donor-specific”) or from another individual (“third party”). For MSCs (which are by far the most commonly used stem cell therapy in clinical trials to date), pre-clinical investigation of the immunogenicity of allo-MSCs in animals has indicated the potential to induce both donor-specific immune hypo-responsiveness and donor-specific allo-antibodies.^102–104^ Such a duality of immunological effects is also evident in human trials of allo-MSCs, in which signals of therapeutic benefits and immune modulation have, in some cases, been accompanied by increases in anti-HLA antibodies.^66^^,^^67^^,^^87^^,^^91^ These observations are especially important in the area of kidney disease in which the induction of anti-HLA antibodies constitutes a significant barrier to future transplantation or to long-term survival of an existing KTx. Thus, the incorporation of detailed longitudinal immunological monitoring protocols and tissue-level analyses of inflammation into controlled clinical trials of allogeneic stem therapies remains critical to understanding their long-term safety and efficacy.

There is a range possible reasons why the therapeutic effects of stem cells that have repeatedly been observed in animal models of kidney diseases and KTx have not seamlessly translated into beneficial immune modulatory/anti-inflammatory effects in human trial subjects. Clearly, species-specific differences in disease pathogenesis and immune responses represent an important barrier to translation of cell-based therapies^105^—highlighting the potential for humanized in vivo and in vitro models to be used to refine or complement the results of animal experiments.^106^ Other key variables also pose greater difficulty to the clinical translation of cell therapies compared to pharmaceutical agents. These include identifying the optimal dose range and frequency, selecting the most effective route of administration, quantifying the persistence of administered cells and their downstream immunological effects, and defining “responder” phenotypes among eligible patients.^43^^,^^107^ Accounting for these details effectively within the design of a cell therapy clinical trial represents a major challenge to the field that is closely linked to the lack of a single, defined mechanism of action for most stem cell therapies which can be measured for each manufactured batch to ensure therapeutic potency.^43^ Furthermore, in the absence of assays that precisely quantify the potency of a stem cell product to deliver its therapeutic effect for a specific disease target, other variables that are inherent to the process of manufacturing cells—such as donor-to-donor heterogeneity, culture medium and conditions, number of cell doublings, and cell cryopreservation/reconstitution protocol—also remain difficult to standardize.^107^ In critically appraising the current level of understanding of the immunological effects of stem cell therapies for kidney diseases and KTx alongside the body of clinical trial results that have been reported to date, we note the great diversity of individual cell products and administration protocols as well as the relatively limited continuity of mechanistic validation between pre-clinical and clinical phases. While understandable, this “broad but shallow” evidence base speaks to the need for more focused collaboration and innovation among those developing stem cell therapies and designing trials for specific kidney diseases.

Finally, it is important to consider that immune modulatory stem cell therapies which show promise for clinical applications to kidney diseases and KTx will ultimately be required to meet diverse regulatory standards and to demonstrate practical and economic feasibility across different healthcare systems if they are to fulfil their potential to reduce the burden of kidney disease at a global scale.^43^^,^^108^ In this regard, the achievements of the investigators in many different countries who have acquired ethical and regulatory approval to conduct first-in-human and early-phase clinical trials of GMP-grade stem cells therapies should not be underestimated. Specifically, these trials and the research studies associated with them have broadly established the feasibility of safely delivering such therapies to patients with complex kidney diseases and have demonstrated the potential for clinical benefits. If these benefits are subsequently proven to be consistent and durable, it is likely that they will be considered cost effective by healthcare payors compared to current life-long pharmacological regimens despite high per-dose pricing.^108^^,^^109^

## Conclusions

As we review here, basic and pre-clinical investigations have delivered new depths of understanding of immune and inflammatory pathways that drive the progression of kidney diseases and KTx complications as well as of the mechanisms and therapeutic strategies by which these pathways can be modulated by stem cell therapies. With the exception of the use of HSCT to successfully induce donor-specific immune tolerance in KTx recipients, translation of this knowledge base to the clinical arena has primarily generated promising but, as yet, non-definitive evidence of clinical benefits and associated immune modulatory/anti-inflammatory effects of auto- or allo-MSCs and an autologous kidney cell-derived product. Renal progenitor cells, iPSC-derived stem cells and stem cell-derived EVs also look set to enter the clinical arena in similar fashion in the coming years. The goal of bridging the gap from this current level of translation to the use of clinically validated stem cells therapies in routine practice will require us to overcome several important bottlenecks and associated challenges which are discussed above and summarized along with possible solutions in Table 2.

**Table 2.** Key bottlenecks and associated challenges to the clinical validation of immune modulatory stem cells therapies for routine use in kidney disease and kidney transplantation.

Key Bottleneck	Associated challenges	Potential solutions
*Lack of potency assays linked to disease-specific therapeutic mechanism of action (MoA) of stem cell therapies*	Differences in disease pathogenesis and immune responses between animal models and human patients Donor to donor variability of source cells Heterogeneity introduced by culture conditions and number of cell doublings Functional changes due to cryopreservation, storage and thawing/re-constitution Difficult pathway to regulatory and marketing approval	Application of humanized in vitro and in vivo models of disease to validate MoA of stem cells Use of single cell and spatial tissue analyses to more precisely define primary mechanistic pathways Stem cell modification by synthetic biology/gene enhancement to optimize defined MoA Development of assays that directly quantify potency of stem cells to deliver defined MoA for specific indications
*Unclear distribution, persistence and duration of immunological effects of stem cell therapies*	Difficulty optimising cell dose, dose frequency, and route of administration Lack of precise confirmation of therapeutic immune modulatory effects in recipients Persistent concerns about immunogenicity or long-term adverse effects Difficult pathway to regulatory and marketing approval	Application of clinically relevant, MoA-specific potency assays and outcomes to pre-clinical models Development of mechanistically informed stem cells in vivo tracking and tissue-level immune profiling methods in humans Incorporation of standardized assays of immune modulation and immunogenicity into clinical trials of stem cell therapies
*Broad range of Phase 1/2 clinical trials of diverse design and quality with very limited progression to robust Phase 3 trials*	Non-definitive evidence of therapeutic efficacy Lack of robust comparisons with “current best practice” and of proven strategies for integration into standard care Limited understanding of patient “responder” phenotypes Unclear cost effectiveness and path to market	Inter-institutional and international collaboration/innovation on stem cell trial design and outcomes analysis Development of assays to identify and quantify MoA-specific responses in recipients of stem cell therapies for kidney diseases Increased focus on health economic analyses of stem cell therapy clinical trials
*Lack of international standards for characterizing safe and therapeutically active stem cell products*	Poor comparability among stem cell products and clinical trial results Lack of opportunities to develop improvements and efficiencies in the manufacturing process Lack of international, multi-center clinical trials of stem cell therapies Difficult pathway to regulatory and marketing approval	Increased international collaboration on stem cell potency validation, product definition, trial design, and outcomes analysis Innovation in stem cells manufacturing guided by MoA-specific potency assays

It is noteworthy that basic research continues to reveal new mechanistic details which highlight the distinct immune modulatory potential of stem cell therapies for kidney disease. For example, Zhang et al. demonstrated that IV BM-MSCs reduced DKD severity in association with reduced number and maturation of CD103^+^ DCs and CD8 T cell activation.^110^ Additionally, MSCs were shown to tune the inflammatory microenvironment by modulating the mTOR-mediated equilibrium between pro-inflammatory Th17 cells and anti-inflammatory Treg, protecting injured renal tubular epithelial cells–a mechanism that may be particularly relevant to AKI and prevention of kidney transplant rejection.^111^ In a mouse model of allogeneic KTx, Xie et al. demonstrated that IV auto-BM-MSCs resulted in reduced severity of acute rejection in associated with increased APRIL phosphorylation and promotion of IL-10^+^ regulatory B cells.^112^ The long-term promise of the field may well lie in harnessing emerging technologies in synthetic biology, gene modification and controlled differentiation^113^ to develop potent, precisely-defined stem cell therapies and translational strategies that more precisely replicate such mechanisms to the benefit of people living with kidney disease and kidney failure.

## Data sharing plan

No new data were generated or analyzed in support of this review article
